# Attention-aided lightweight networks friendly to smart weeding robot hardware resources for crops and weeds semantic segmentation

**DOI:** 10.3389/fpls.2023.1320448

**Published:** 2023-12-21

**Authors:** Yifan Wei, Yuncong Feng, Xiaotang Zhou, Guishen Wang

**Affiliations:** ^1^ College of Computer Science and Engineering, Changchun University of Technology, Changchun, Jilin, China; ^2^ Artificial Intelligence Research Institute, Changchun University of Technology, Changchun, Jilin, China; ^3^ Key Laboratory of Symbolic Computation and Knowledge Engineering of Ministry of Education, Jilin University, Changchun, Jilin, China

**Keywords:** convolutional neural network, attention mechanism, lightweight semantic segmentation, crop and weed segmentation, precision farming

## Abstract

Weed control is a global issue of great concern, and smart weeding robots equipped with advanced vision algorithms can perform efficient and precise weed control. Furthermore, the application of smart weeding robots has great potential for building environmentally friendly agriculture and saving human and material resources. However, most networks used in intelligent weeding robots tend to solely prioritize enhancing segmentation accuracy, disregarding the hardware constraints of embedded devices. Moreover, generalized lightweight networks are unsuitable for crop and weed segmentation tasks. Therefore, we propose an Attention-aided lightweight network for crop and weed semantic segmentation. The proposed network has a parameter count of 0.11M, Floating-point Operations count of 0.24G. Our network is based on an encoder and decoder structure, incorporating attention module to ensures both fast inference speed and accurate segmentation while utilizing fewer hardware resources. The dual attention block is employed to explore the potential relationships within the dataset, providing powerful regularization and enhancing the generalization ability of the attention mechanism, it also facilitates information integration between channels. To enhance the local and global semantic information acquisition and interaction, we utilize the refinement dilated conv block instead of 2D convolution within the deep network. This substitution effectively reduces the number and complexity of network parameters and improves the computation rate. To preserve spatial information, we introduce the spatial connectivity attention block. This block not only acquires more precise spatial information but also utilizes shared weight convolution to handle multi-stage feature maps, thereby further reducing network complexity. The segmentation performance of the proposed network is evaluated on three publicly available datasets: the BoniRob dataset, the Rice Seeding dataset, and the WeedMap dataset. Additionally, we measure the inference time and Frame Per Second on the NVIDIA Jetson Xavier NX embedded system, the results are 18.14 msec and 55.1 FPS. Experimental results demonstrate that our network maintains better inference speed on resource-constrained embedded systems and has competitive segmentation performance.

## 1 Introduction

Weeds pose a significant challenge to global agriculture due to their short growth cycle and rapid reproduction rate. They fiercely compete with crops for resources such as water, sunlight, and soil nutrients, ultimately leading to reduced crop yield and compromised quality (Hasan et al., 2021). Early detection of weeds in the field is highly critical for taking appropriate actions. Conventional weed control employs two primary methods. The first is mechanized weed control, which is typically employed in weed-concentrated areas and allows for the selection of appropriate tools to remove them. Nevertheless, mechanical weeding becomes cost-prohibitive for extensive planting fields. The second method is chemical control, which relies on the application of herbicides to target and eliminate weeds while preserving the crop (Rakhmatulin et al., 2021). Chemical weed control is the predominant approach due to its advantages in saving time and labor, convenience, and high efficiency. Nonetheless, uneven herbicide spraying can lead to insufficient weed control in areas with concentrated weed growth, resulting in wasted herbicides, excessive chemical residues, and even crop damage or loss (Kudsk and Streibig, 2003).

To overcome the limitations of chemical weed control methods, (Slaughter et al., 2008) proposed an automated weed detection system employing smart agricultural technology. The primary objective of this system is to attain precise herbicide application specifically on weeds, ultimately reducing expenses and minimizing chemical usage. The persistent challenge of distinguishing between crops and weeds, coupled with the complexities of background interference, has been a longstanding issue. However, deep learning has revolutionized automation in precision agriculture by significantly enhancing target recognition accuracy and anti-jamming capabilities (Albanese et al., 2021). To empower intelligent weeding robots capable of accurately identifying the target weeds and executing effective autonomous weed control, achieving precise segmentation of crops and weeds becomes absolutely crucial (Jiang and Li, 2020; Yang and Xu, 2021).

The Deep Neural Networks (DNNs) have been widely employed in semantic segmentation in recent years (Krizhevsky et al., 2012). (You et al., 2020) have augmented DNN with the attention mechanism, which has shown promising results in crop and weed segmentation tasks. Nevertheless, due to the significant number of network parameters and high complexity, DNNs equipped in embedded systems may result in poor inference time and low Frame Per Second (FPS). This drawback renders them ill-suited for deployment in weeding robots characterized by constrained hardware resources. Hence, it is imperative to develop lightweight networks specifically designed for the semantic segmentation of crops and weeds (Rai et al., 2023). The Convolutional Neural Network (CNN) is an effective method for designing lightweight semantic segmentation networks with a wide range of applications (Ji et al., 2020; Wang et al., 2021). Numerous CNN networks specifically designed for autonomous driving (Romera et al., 2017; Wu et al., 2020), medical images (Ronneberger et al., 2015), or general-purpose segmentation tasks (Long et al., 2015; Zhao et al., 2017) have found application in the domain of crop and weed segmentation as well. (Wu et al., 2021) examined the crop and weed detection and segmentation, and provided the results of comparison with previously proposed general-purpose CNN models. The study confirmed that CNNs exhibit high performance in accurate crop and weed recognition and segmentation.

However, most current CNN-based semantic segmentation networks for crop and weed focus solely on segmentation accuracy, disregarding practical application. This limitation hinders their effective implementation on embedded systems. Although the CNN-based general-purpose lightweight network has indeed shown enhanced inference time and FPS on embedded systems, its segmentation accuracy falls short of meeting practical requirements for crop and weed segmentation. Considering the disadvantages in previous studies, we proposed an attention-assisted lightweight network for semantically segmenting crops and weeds, and achieves a robust balance between network complexity and segmentation performance. The main contributions of this study are summarized as follows.

1. The proposed method not only boasts a low parameter count and minimal complexity but also achieves favourable inference time and FPS when implemented on embedded systems. This makes it particularly well-suited for weeding robots that have limited hardware resources. Furthermore, it holds a competitive edge in terms of segmentation performance.
2. The dual attention block (DA) is proposed for datasets with limited samples and a high degree of similarity, specifically designed for crop and weed datasets. The DA allows for the exploration of potential relationships across the entire dataset, offering robust regularization to overcome the issue of overfitting during training. Additionally, it enhances the generalization ability of the attention mechanism by integrating information between channels.
3. The refinement dilated conv block (RDC) is proposed to address the challenges associated with plant (including crop and weed) and background segmentation, as well as the difficulty in classifying the semantic information of weed and crop. Positioned as a replacement for 2D convolution within the high-level encoder, the RDC module promotes the fusion of global and local information, strengthens the integration of multiscale data, and concurrently trims down the number of network parameters.
4. The spatial connectivity attention block (SCA) is presented to address the challenge posed by the small size of weed targets and the potential loss of spatial information resulting from the convolutional layers’ superimposition. The SCA not only reduces the loss of spatial information but also enhances the accuracy of spatial information. Additionally, it employs weight sharing instead of the complex connection between the encoder and decoder, contributing to a further reduction in network complexity.

The remaining parts of this paper are organized as follows. Section 2 illustrates the related works. Section 3 details the proposed method. Section 4 presents the performance comparison and analysis of the proposed and state-of-the-art (SOTA) methods. Section 5 discusses the experimental results of different algorithms comprehensively. The conclusions are drawn in Section 6.

## 2 Related works

In recent years, CNNs have risen to prominence as powerful deep learning algorithms for image visualization and pattern recognition, necessitating minimal human intervention (Pouyanfar et al., 2018). This extensive capacity has facilitated the adoption of CNNs across various domains of computer vision. Particularly in agriculture, CNNs have been extensively utilized to address diverse challenges and have become the primary approach in computer-aided solutions for agricultural production issues (Kamilaris and Prenafeta-Boldu, 2018).

Weed control is a significant challenge in agricultural production. Weeds have a significant and profound impact on crop growth. The weed-related challenges through computer-aided solutions mainly include (1) image classification for distinguishing crops from weeds, (2) target detection to identify crops and weeds, and (3) pixel-by-pixel semantic segmentation for precise delineation of crops and weeds. (Pandey and Jain, 2022) proposed a novel conjunctive dense CNN architecture for image classification of multiple crop and weed classes from RGB images captured by UAV (unmanned aerial vehicle). However, this image classification network is insufficient for solving weed problems as it cannot simultaneously identify multiple classes. Regarding target detection of crops and weeds, (Wang et al., 2022) constructed a YOLOCBAM model aimed at enhancing its predictive capabilities by effectively eliminating irrelevant features through the use of a convolutional block attention module (CBAM) (Woo et al., 2018). (Chen et al., 2022) have also incorporated spatial and channel attention in YOLOv4 to increase the recognition accuracy of weeds. Nevertheless, the CNN-based target detection network fails to accurately determine crop and weed boundaries due to its rectangular boundary detector. In weed-rich areas, occlusions and overlaps between crops and weeds highly affect target detection accuracy, making it unsuitable for weed problemsolving. Regarding pixel-by-pixel semantic segmentation of crops and weeds, (You et al., 2020) have made improvements to CNN-based models by employing techniques such as extended convolution (Zhen et al., 2019) and multiscale approaches (Chen et al., 2017b). These efforts have led to consistent improvements in segmentation accuracy. Likewise, adding specific modules to mature CNN networks significantly enhances crop and weed semantic segmentation performance. Comparing image classification and target detection with pixel-by-pixel semantic segmentation, the latter method allows for precise target detection and accurate boundary delineation. This capability is crucial for precision herbicide spraying operations of intelligent weeding robots. Consequently, we choose to build a CNN-based crop and weed semantic segmentation network for computer-aided solutions for weed problem-solving.

CNN-based crop and weed semantic segmentation networks can be classified into two categories: single-CNN-based methods and multi-CNN-based methods. Within the multi-CNN-based methods, (Khan et al., 2020) introduced a cascaded encoder-decoder architecture that divides the training into multi-stage multitasks. Although this approach enhances the segmentation accuracy, it fails to meet practical requirements. To address these limitations, (Kim and Park, 2022) proposed a single-stage processing method where target segmentation is performed simultaneously with crop and weed segmentation, and (Moazzam et al., 2023) optimizes it to have fewer parameters. This approach facilitates practical. Among the single-CNN-based methods, Enet (Paszke et al., 2016), DeepLab (Chen et al., 2014) and its variant networks (Chen et al., 2018), as well as the common symmetric encoder-decoder networks UNet (Ronneberger et al., 2015) and SEGNet (Badrinarayanan et al., 2017), are widely utilized for crop and weed semantic segmentation tasks. However, these networks are not specifically designed for crop and weed semantic segmentation tasks but rather belong to general-purpose networks. In recent years, (Janneh et al., 2023) have developed specialized networks that significantly improve the segmentation performance compared to general-purpose networks for crop and weed semantic segmentation tasks. To be deployed in intelligent weeding robots, these networks must not only demonstrate excellent segmentation performance but also possess a low parameter count and minimal complexity. Additionally, they should be compatible with embedded systems, ensuring improved inference time and FPS.

Since the number of parameters and inference time multiple increases with multi-CNNs, the single-CNN model is utilized in this paper. The dominant network structure among current single-CNN methods is based on the encoder-decoder model. The encoder extracts semantic information about the crops and weeds at the expense of spatial information, while the decoder up-samples the features to regain the lost spatial information. Based on the enhancement of the symmetric encoder-decoder structure, numerous network architectures have been proposed. (Valanarasu and Patel, 2022) combined a multi layer perceptron (MLP) with the UNet, yielding a significant reduction in network parameters and complexity. This innovative approach successfully preserves segmentation performance, while concurrently optimizing inference time and FPS. Furthermore, incorporating an attention-based mechanism into the encoder and decoder structure can substantially enhance performance and stability. (Guo et al., 2022) added External Attention to the network, which improves the network’s ability to extract information from the entire dataset. (Cao et al., 2019; Wang et al., 2020) introduced Spatial Attention and Channel Attention, leading to a significant enhancement of the network’s ability to extract relevant information while suppressing irrelevant information. In this paper, we optimize these methods by tailoring them to the specific requirements of crop and weed segmentation tasks, and then integrate them into a foundational six-stage lightweight U-shaped structure. As a result, the network achieves a reduction in parameters and complexity, enhances inference time, and attains a higher FPS when operating on embedded systems, all while maintaining a competitive segmentation performance.

## 3 The proposed method

### 3.1 The framework of the proposed method

**Figure 1:**
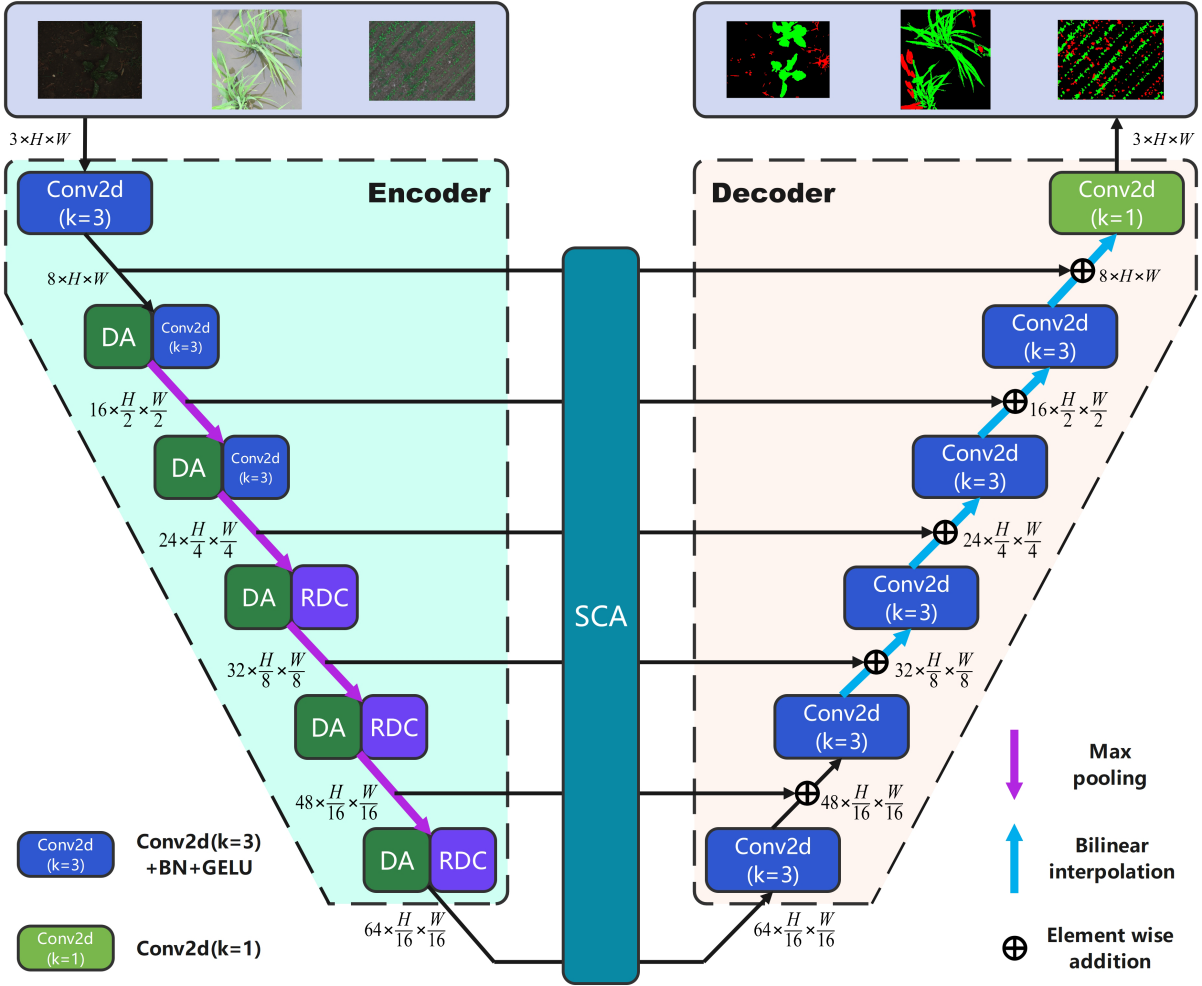
The framework of the proposed network.

In this paper, we propose a lightweight baseline comprising a symmetric encoder and decoder structure with a six-stage U-shaped structure. At each stage, the channels are assigned as {8,16,24,32,48,64}, and skip connections are established between them through element-wise addition. **Figure 1** displays the framework of the proposed network.

As shown in **Figure 1**, the encoder part consists of six stages. In the stage1, feature extraction is performed using the Conv2d block (2D convolution ( kernel=3,stride=1 ) + batch normalization + GELU activation function). In subsequent stages (2 to 6), we introduce the dual attention block and max pooling ( stride=2 ) for downsampling, which enhances the regularization and attention mechanisms, while also incorporating channel information. In stages three to six, we utilize the refinement dilated conv block instead of the Conv2d block for further feature extraction. This allows for the integration of both local and global information while simultaneously reducing the number of network parameters. Moving on to the decoder part, we employ the Conv2d block and bilinear interpolation to perform up-sampling and recover the feature information. Additionally, we introduce the spatial connectivity attention block during the skip connection between the encoder and decoder. We store the feature information of the encoder outputs in a list and then utilize dilated convolution with shared weights to process the multi-scale features. This approach effectively reduces the loss of spatial information, resulting in more accurate spatial information retrieval and decreased network complexity. Finally, after the Conv2d block [2D convolution ( kernel=3,stride=1 )], the number of output channels is adjusted to obtain the segmentation results. By integrating the aforementioned three blocks with a lightweight baseline, our study enhances segmentation performance while minimizing the additional burden on the complexity of network parameters. In the following sections, we provide a detailed description of these modules.

### 3.2 Dual attention block

Self attention (SA) is one of the prominent attention mechanisms in computer vision tasks (Dosovitskiy et al., 2020). It can effectively capture long-distance dependencies by computing the correlation between all positions within a sample. (Jiang et al., 2022; Reedha et al., 2022; Zhang et al., 2023a) have added SA to the network to apply it to crop and weed detection and segmentation tasks. However, SA possesses high computational complexity and does not address the inter-sample connection, which contradicts the original objective of designing a lightweight network in this paper. To address these limitations, we employ external attention (EA), which exhibits lower computational complexity. Unlike SA, EA utilizes two separate external storage memory units to compute the attention of input samples. They are defined as (Equation 1, 2).


(1) $$X=X_{In}⊗M_{k}^{T},$$

(2) $$X_{Out}=Softmax\left(X\right)⊗M_{V},$$

where $X_{In}\in {\mathbb{R}}^{N\times C}$ , *C* denotes the number of channel dimensions, *N* (*N* = *H* × *W*) represents the total number of pixels in the feature map. *M_K_*, $M_{V}\in {\mathbb{R}}^{4C\times C}$ denote two separate external storage memory units, respectively. *X* is the feature map derived by learning the prior knowledge of the input samples and updating the features by the similarity of *X* with *M_K_* and *M_V._* ⊗ denotes matrix multiplication.

**Figure 2:**
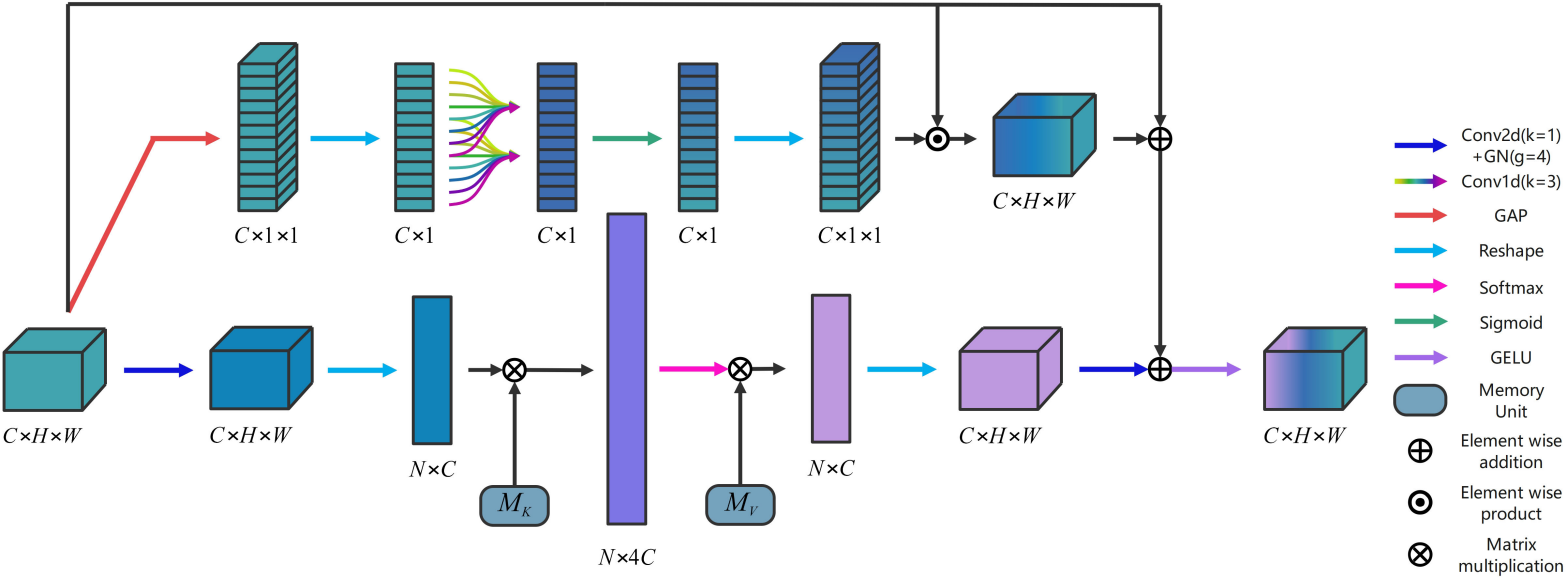
The architecture of the proposed DA.

The memory units finally transform the input into a higher-dimensional space, enabling it to comprehensively depict the overall feature information of the dataset. To effectively capture the inherent relationships within the dataset, we proposed a novel dual attention block (DA) based on EA is illustrated in **Figure 2**. The fast 1D convolution ( kernel=3,stride=1 ) is employed to enable all channels to share the same set of learning parameters. The feature maps following the inclusion of local cross-channel interactions are incorporated into the attention model as residual connections of the EA mechanism. Then, a DA is formed. This approach effectively captures the inherent relationships within the entire dataset, and provides robust regularization. Moreover, DA enhances the generalization capability of the attention mechanism, and facilitates information interaction between the channels, leading to improved integration of information. The specific implementation process of DA is illustrated as follows.

Given an input $X_{In}\in {\mathbb{R}}^{C\times H\times W}$ , there are two branches in the DA. In the first branch, the output $X_{Out1}\in {\mathbb{R}}^{C\times H\times W}$ is computed by (Equation 3).


(3) $$\;X_{Out1}=C_{2D}\left(R\left(M_{V}⊗Softmax\left(X_{1}\right)\right)\right),$$

where $C_{2D}$ denotes the 2D convolution ( kernel=1,stride=1 ) + group normalization ( groups=4 ). R denotes the Reshape operation. $M_{V}\in {\mathbb{R}}^{4C\times C}$ denotes the memory cell. Softmax denotes the softmax activation function. ⊗ denotes the matrix multiplication. $X_{1}\in {\mathbb{R}}^{N\times 4C}$ is computed by as (Equation 4).


(4) $$X_{1}=R\left(C_{2D}\left(X_{In}\right)\right)⊗M_{K}^{T},$$

where $M_{K}^{T}\in {\mathbb{R}}^{4C\times C}$ denotes the memory cell. In the second branch, the output $X_{Out2}\in {\mathbb{R}}^{C\times 4C}$ is calculated by (Equation 5).


(5) $$X_{Out2}=R\left(\sigma \left(X_{2}\right)\right)⊙X_{In},$$

where R denotes the Reshape operation. σ denotes Sigmoid activation function. ⊙ denotes the element-wise product. $X_{2}\in {\mathbb{R}}^{C\times 1}$ is computed by (Equation 6).


(6) $$X_{2}=C_{1D}\left(R\left(G\left(X_{In}\right)\right)\right),$$

where $C_{1D}$ denotes the standard 1D convolution ( kernel=3,stride=1 ). G represents the global average pooling. The final output $X_{Out}\in {\mathbb{R}}^{C\times H\times W}$ is computed by (Equation 7).


(7) $$X_{Out}=GELU\left(X_{Out1}⊕X_{Out2}⊕X_{In}\right),$$

where ⊕ denotes the element-wise addition. GELU denotes the *GELU* activation function (Hendrycks and Gimpel, 2016).

### 3.3 Refinement dilated conv block

**Figure 3:**
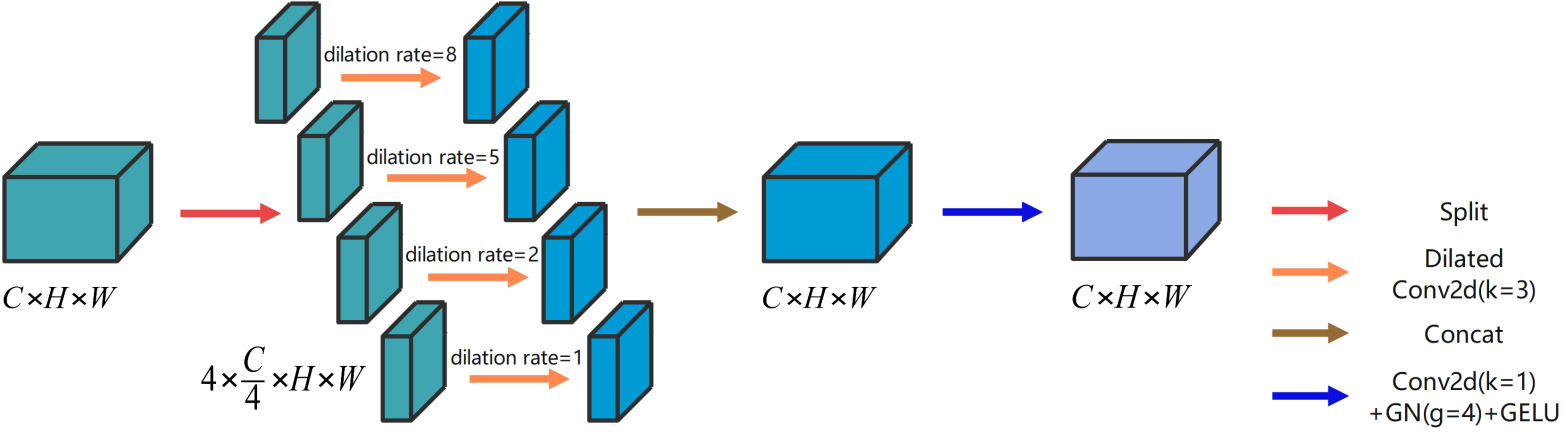
The architecture of the proposed RDC.

Both local and global information are crucial for improving segmentation performance. On the one hand, global information assists the network in comprehending the overall plant structure and background, thus enabling more precise localization of the segmentation target region. On the other hand, local information helps capture the edge details of the crop and weeds, enhancing segmentation accuracy. One common approach to obtain global information is to increase the receptive field using 2D convolution with large convolution kernels. However, the simultaneous acquisition of both local and global information necessitates the concurrent utilization of 2D convolution with convolution kernels of different sizes. This approach substantially inflates the number of parameters. To address this issue, we propose the refinement dilated conv block (RDC) that leverages dilated convolutions with different dilation rates in parallel. Dilated convolution enables enlargement of the receptive field without increasing the number of parameters (Chen et al., 2017a), while maintaining the size of the output feature mapping (Yu and Koltun, 2015). **Figure 3** illustrates the architecture of the proposed RDC.

The RDC includes four types of dilation convolutions with distinct dilation rates. The dilation convolutions with dilation rates of 1 and 2 extract local information, whereas the dilation convolutions with dilation rates of 5 and 8 extract global information. We can obtain precise output results by aggregating the multiscale information. Additionally, we introduce a channel dimension splitting operation that divides the input into four parts, each corresponding to a dilation convolution with a different dilation rate. This allows us to cover the segmentation target from the smallest to the largest region, further improving the computation rate of the convolution. The specific implementation process of RDC is illustrated as follows.

Given an input $X_{In}\in {\mathbb{R}}^{C\times H\times W}$ , it is firstly divided into four parts $x_{i}\in {\mathbb{R}}^{\frac{C}{4}\times H\times W}(i=1,2,3,4)$ along the channel dimension, as shown in (Equation 8).


(8) $$\left(x_{1},x_{2},x_{3},x_{4}\right)=S\left(X_{In}\right),$$

where S denotes the Split operation. Then the dilation convolution ( kernel=3,stride=1 ) with different dilatation rates is performed to obtain the four parts $x_{i}^{{\;}^{'}}\in {\mathbb{R}}^{\frac{C}{4}\times H\times W}\left(i=1,2,3,4\right)$ , as shown in (Equation 9).


(9) $$\left(x_{1}^{{\;}^{'}},x_{2}^{{\;}^{'}},x_{3}^{{\;}^{'}},x_{4}^{{\;}^{'}}\right)=DC_{i}\left(x_{1},x_{2},x_{3},x_{4}\right)\left(i=1,2,5,8\right)$$

where $DC_{i}$ denotes dilation convolution ( kernel=3,stride=1 ) with different dilation rates and i is the dilation rate. The final output $X_{Out}\in {\mathbb{R}}^{C\times H\times W}$ is calculated by (Equation 10).


(10) $$X_{Out}=C_{2D}\left(C_{on}\left(x_{1}^{{\;}^{'}},x_{2}^{{\;}^{'}},x_{3}^{{\;}^{'}},x_{4}^{{\;}^{'}}\right)\right),$$

where $C_{2D}$ denotes the 2D convolution ( kernel=1,stride=1 ) + group normalization ( groups=4 ) + GELU activation function. $C_{on}$ denotes the Concat operation.

### 3.4 Spatial connectivity attention block

Spatial attention plays a crucial role in suppressing irrelevant information and acquiring precise spatial information. The CNN structure often leads to a loss of spatial information due to the combining effects of convolutional layers. To tackle this issue, (Woo et al., 2018) have introduced a spatial attention mechanism to the network, enabling it to identify the most critical regions for further processing. This approach helps mitigate the loss of spatial information and improve the accuracy of spatial information retrieval.

In the specific context of crop and weed semantic segmentation, the weed information is relatively scarce, resulting in an increased risk of losing spatial information. Therefore, incorporating a spatial attention mechanism into the network becomes imperative. In this paper, we propose the spatial connectivity attention block (SCA) that facilitates multi-stage parallel connections to generate spatial attention feature maps.

**Figure 4:**
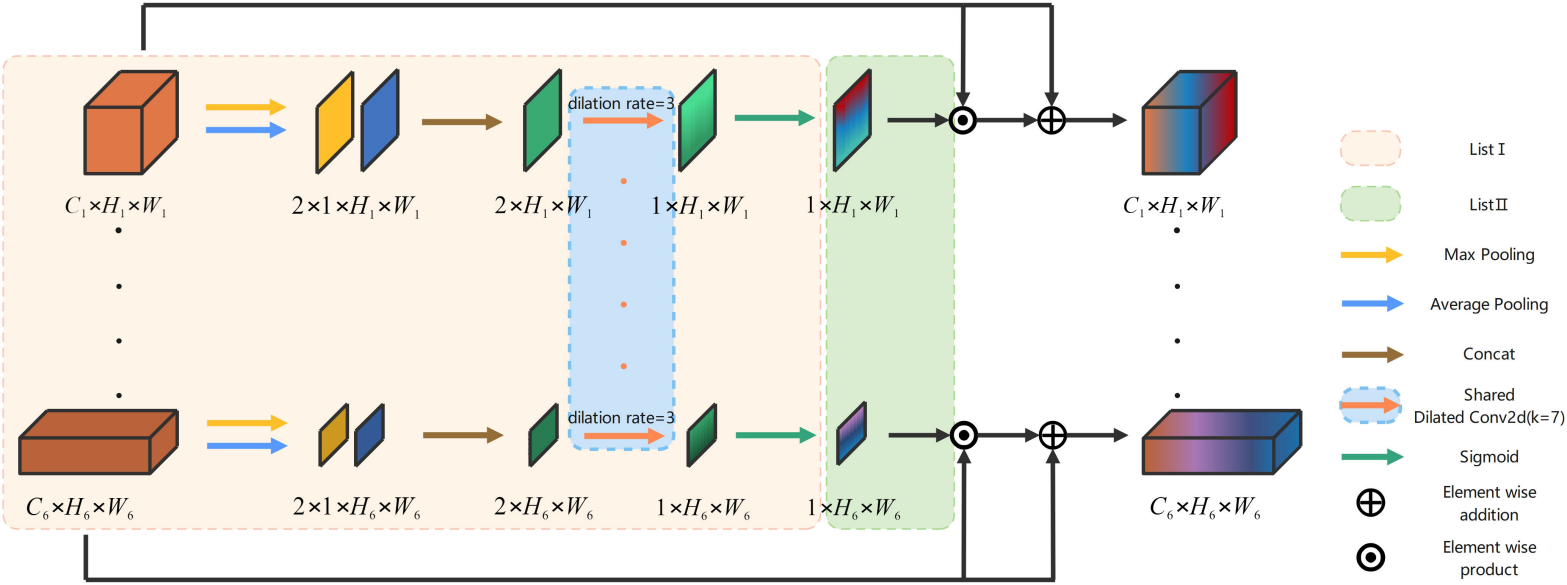
The architecture of the proposed SCA.


**Figure 4** gives the architecture of the proposed SCA, and it is an example of six-stage encoder-decoder body structure. Stages 2, 3, 4, and 5, which are shown omitted in **Figure 4**, are computed using the same method as stages 1 and 6. The outputs from the encoders at each stage are stored in *List*I and subsequently utilized to calculate average pooling and maximum pooling operations based on the channel dimensions. These results are then concatenated separately, as illustrated in (Equations 11, 12).

(11) $$A=ListI[X_{1},X_{2},X_{3},X_{4},X_{5},X_{6}],$$

(12) $$B_{i}=Concat\left(Maxpool\left(A\right);Avgpool\left(A\right)\right)\left(i=\;1,2,3,4,5,6\right)$$

Convolution with shared weights can significantly reduce network parameters and complexity. In SCA, a dilated convolution ( kernel=7,stride=1 ) with a dilation rate of 3 and a Sigmoid activation function are employed to generate the spatial attention feature maps *P* for the encoders at each stage. These maps are then incorporated into the variable *List*II according to (Equations 13, 14).


(13) $$P_{i}=Sigmoid\left(DliatedConv2d\left(B_{i}\right)\right)(i=\;1,2,3,4,5,6),$$

(14) $$T=ListII\;=\;[P_{1},P_{2},P_{3},P_{4},P_{5},P_{6}],$$

The SCA holds crucial significance in mitigating spatial information loss and enhancing the precision of spatial information acquisition. The utilization of a parallel connection with weight sharing effectively diminishes the intricate connection between the encoder and decoder, thereby considerably reducing both the quantity and complexity of network parameters. The specific implementation process of SCA is illustrated as follows.

Given output of the encoder of each stage as the inputs $X_{Ini}\in {\mathbb{R}}^{Ci\times Hi\times Wi}\left(i=\;1,2,3,4,5,6\right)$ at each stage of the SCA, they are firstly placed in *List*I, as shown in (Equation 15).


(15) $$A=ListI[X_{In1},X_{In2},X_{In3},X_{In4},X_{In5},X_{In6}]$$

then we traverse *A* to obtain two feature maps $x_{i}1,x_{i}2\in R^{1\times Hi\times Wi}\left(i=\;1,2,3,4,5,6\right)$ at each stage after max pooling and average pooling in channel dimension. Concat along the channel dimension to obtain $X_{i}{-}_{1}\in {\mathbb{R}}^{2\times Hi\times Wi}\left(i=\;1,2,3,4,5,6\right)$ is calculated by (Equation 16).


(16) $$X_{i}{-}_{1}=C_{on}\left(MP_{C}\left(A\right),AP_{C}\left(A\right)\right)\left(i=\;1,2,3,4,5,6\right)$$

where *C_on_* denotes the Concat operation. *MP_C_* and *AP_C_* denotes the channel-based max pooling and average pooling, respectively. Next, the spatial attention feature map $X_{SAi}\in {\mathbb{R}}^{1\times Hi\times Wi}\left(i=\;1,2,3,4,5,6\right)$ for each stage is calculated by (Equation 17).


(17) $$X_{SAi}=\sigma (DC(X_{i}{-}_{1}))(i=\;1,2,3,4,5,6),$$

where *σ* denotes Sigmoid activation function, and *DC* denotes the shared dilated convolution (*kernel* = 7*,stride* = 1*,rate* = 3). Then, all the spatial attention feature maps are placed in *List*II, as shown in (Equation 18).


(18) $$T=ListII\;=\;[X_{SA1},X_{SA2},X_{SA3},X_{SA4},X_{SA5},X_{SA6}],$$

the final output $X_{Outi}\in {\mathbb{R}}^{Ci\times Hi\times Wi}\left(i=\;1,2,3,4,5,6\right)$ is computed by (Equation 19).


(19) $$X_{Outi}=\;(T⊙X_{Ini})⊕X_{Ini}\left(i=\;1,2,3,4,5,6\right)$$

where ⊙ denotes the element-wise product, ⊕ denotes the element-wise addition.

## 4 Experimental results and analysis

### 4.1 Datasets

We conducted experiments on three publicly available datasets, including the BoniRob dataset (Chebrolu et al., 2017), the Rice Seeding dataset (Ma et al., 2019), and the WeedMap dataset (Sa et al., 2018). The segmentation task for each dataset involves making pixel-by-pixel predictions of crops and weeds. These datasets are annotated with three types of labels: black, red, and green, which correspond to soil or Paddy background, weeds, and crops, respectively. The limited number of images in the Rice Seeding dataset and WeedMap dataset can easily lead to overfitting and excessive randomness during testing. To address this issue, we employed data augmentation techniques to expand the datasets. It is important to note that if the dataset is expanded first and then divided into sets, data leakage can occur, compromising the significance of the test set. Therefore, in this paper, we divided the dataset into training, validation, and test sets first, and then applied data augmentation techniques to expand the data within each set, ensuring the rigor of our experiments.

(a) BoniRob dataset

The BoniRob dataset utilizes an autonomous robot to gather data from a farm in close proximity to Bonn, Germany, comprising sugar beets and weeds. The images have a resolution of 1296 × 966 pixels. The Bonirob robot collected multiple image datasets due to its diverse array of sensors used for data acquisition. Among the various possibilities, we selected a total of 1050 RGB images and their corresponding color masks in our experiments. The training set consists of 900 images, while the validation set and the test set contain 50 and 100 images, respectively.

(b) Rice Seeding dataset

The Rice Seeding dataset is generated using an IXUS 1000 HS camera (lens model EF-S 36-360 mm f/3.4-5.6 IS STM) in a paddy. The dataset consists of images captured in a paddy, featuring both rice seeding and weeds. The images have a resolution of 912 × 1024 pixels. In total, there are 224 images, which are divided into three subsets: 170 images for training, 18 images for validation, and 36 images for testing. To enhance the dataset, various data augmentation techniques are applied to each subset, including horizontal and vertical flips, vertical and diagonal quadrangle deformations, elastic distortions, and miscut transformations. As a result, the training set was expanded to 850 images, the validation set to 40 images, and the test set to 90 images. In total, the dataset comprises 980 images.

(c) WeedMap dataset

The WeedMap dataset utilizes a UAV platform equipped with multispectral sensors to collect data from two farms located in Switzerland and Germany. The dataset includes imagery of crops and weeds, with an image resolution of 480 × 360 pixels. The entire dataset is divided into seven subsets. To generate these subsets, the original images are processed from upright maps into tiled images using a sliding window approach. However, it is important to note that some of the tiled images may contain regions with invalid pixels after processing. In our experiments, we specifically select the RGB tiled images that do not have any invalid pixel regions as the dataset. In total, there are 245 images, which are divided into three subsets: 200 images for training, 15 images for validation, and 30 images for testing. Since the WeedMap dataset is acquired through drone-based surveillance of farmland, the images exhibit a certain degree of regularity. Consequently, we employ flip-based augmentation techniques (including horizontal flip, vertical flip, and their combinations) to augment the data in the training, validation, and test sets. Each flip method is used only once to ensure that there are no duplicate images in the dataset. As a result, the training set was expanded to 800 images, the validation set to 60 images, and the test set to 120 images. In total, the dataset comprises 980 images.

### 4.2 Implementation details and network training

To simulate the hardware environment of a mobile device, the experiments were conducted on a laptop computer. The laptop was equipped with an AMD Ryzen 9 5900HS CPU, 16GB of RAM, and an NVIDIA GeForce RTX 3050 Laptop GPU. The proposed method is implemented using the default initialization scheme for each module within the PyTorch 2.0 framework. The supervised learning-based models were trained and evaluated using the AdamW optimizer (Loshchilov and Hutter, 2017). The learning rate is reduced by the cosine annealing attenuation method. To safeguard against the loss of the training model due to power outages or abnormal exits during long-term training, we propose a strategy to regularly save the model every 5 epochs. This approach ensures that even in such unfortunate events, the progress made during the training process is preserved (Zhang et al., 2023b).

All the input images are standardized and resized to a size of 256 × 256 pixels, color mode is RGB, and further the data is augmented by applying random Gaussian blur. It’s important to note that the training process did not involve transfer learning with pre-training. We set the learning rate, weight decay, batch size and epochs to 0.001, 0.01, 6 and 150, respectively.

**Figure 5:**
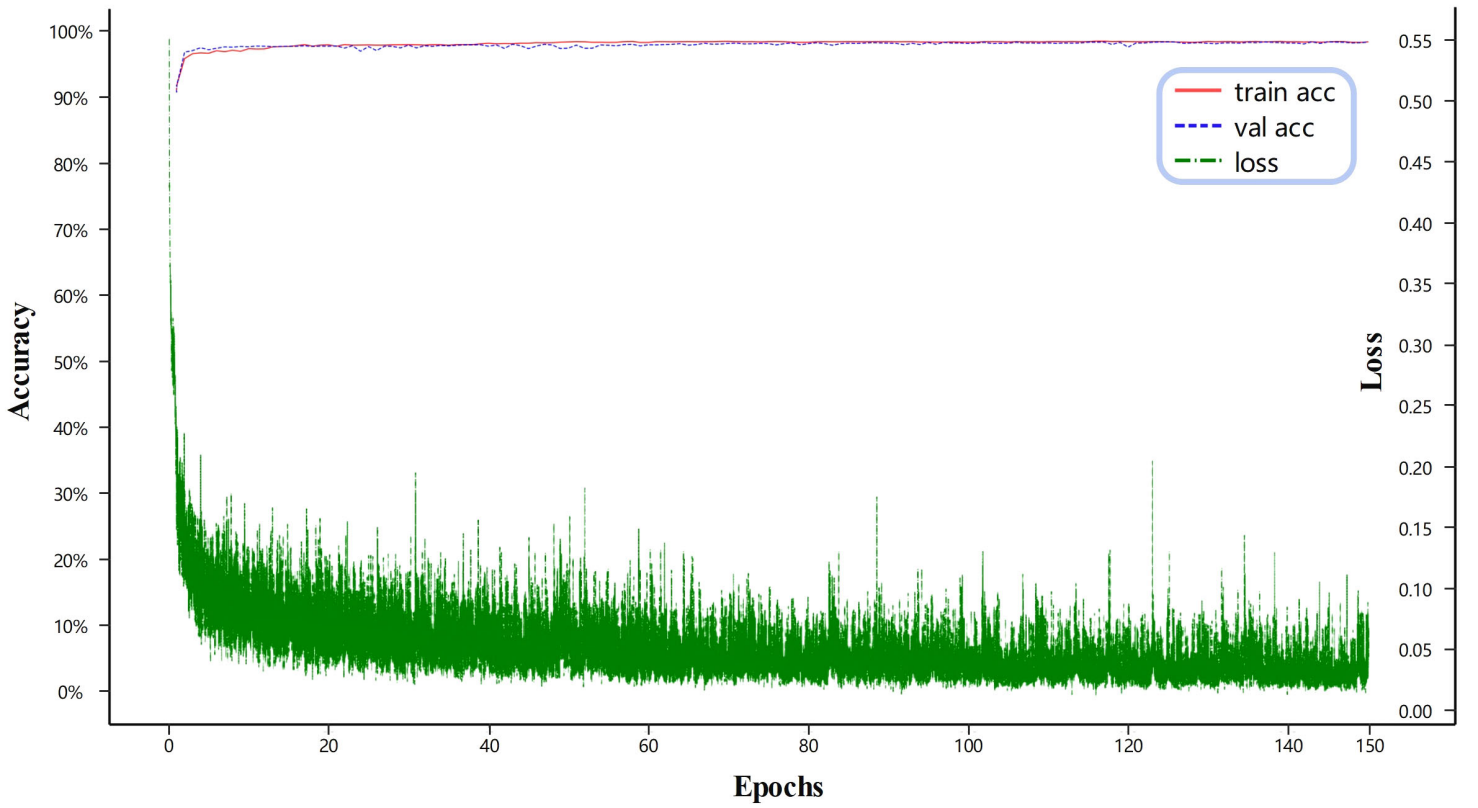
Loss and accuracy of the proposed method on the training and validation sets.

**Figure 5** illustrates the loss and accuracy of the proposed method on both the training and validation sets. The green line represents the loss on the training set, while the red line and the blue line correspond to the accuracy on the training and test sets, respectively. As shown in **Figure 5**, after 80 epochs, the model reaches a state of equilibrium in terms of training loss. Additionally, the training accuracy becomes more similar to the validation accuracy with reduced fluctuations. Looking at the whole picture that when the number of training iterations increases, the loss tends to converge towards sufficiently large scores, while the accuracy converges towards sufficiently small scores. This suggests that the neural network model in this study is effectively trained on the provided training dataset. Similarly, when considering the number of training iterations on the validation dataset, the accuracy eventually reaches a suitably low value. This implies that the model proposed in this study successfully avoids overfitting on the training dataset.

### 4.3 Loss function

The selection of the loss function significantly impacts the learning process, and it is crucial for designing an effective image segmentation architecture. In this study, the main loss function chosen for training the network is Dice loss (Milletari et al., 2016), while Cross-Entropy loss (Loshchilov and Hutter, 2017) is used as an auxiliary loss function. Generally, CNN-based image segmentation relies on capturing high-frequency image distributions. Inadequate representation of high-frequency components can lead to inaccurate region detection. Thus, Dice loss is utilized to enhance the focus on diverse shapes of crops and weeds. Dice loss is commonly utilized in crop and weed segmentation tasks and computer vision, due to its ability to facilitate precise region detection by measuring image similarity. Furthermore, Cross-Entropy loss is utilized to reinforce the training of high-frequency edge component detection in order to complement the overall high-frequency representation. The Dice loss and the Cross-Entropy loss are defined as (Equations 20, 21), respectively.


(20) $$Loss_{Dice}=1-\frac{2\left(\sum_{i=1}^{B\times H\times W}p_{i}\times g_{i}\right)}{\sum_{i=1}^{B\times H\times W}p_{i}^{2}+\sum_{i=1}^{B\times H\times W}g_{i}^{2}},$$

(21) $$\;Loss_{CrossEntropy}=-\sum_{i=1}^{B\times H\times W}p_{i}\text{log }g_{i},$$

where *p_i_* denotes the value of the i-th pixel in the true label, and *g_i_* denotes the value of the i-th pixel predicted by the model. *B*, *H*, *W* denote the batch size, the image height, and the image width, respectively. *B* × *H* × *W* denotes the total number of pixels.

After analyzing the reduction in loss across several training sessions, it is unequivocally determined that the most favorable impact on the learning process is achieved by assigning a weight of 0.8 to the Dice loss and a weight of 0.2 to the Cross-Entropy loss. Consequently, a novel compound loss function is defined in this paper, as depicted in (Equation 22).


(22) $$Loss_{Compound}=0.8Loss_{Dice}+0.2Loss_{CrossEntropy}.$$

### 4.4 Evaluation metrics

To fully measure the performance of our proposed network, we use five objective metrics in the comparison experiments, including Intersection Over Union (IOU) for each class (soil or paddy, crop, and weed), Mean IOU (MIOU), Precision, Recall, and F1score (Yang et al., 2023). Before describing the metrics, some notations will be given first. TP is the number of true positives. FP is the number of false positives. TN is the number of true negative. FN is the number of false negatives.

The IOU is a crucial metric used to assess the accuracy of image segmentation in quantitative terms. We will calculate the IOU value for each class (soil or paddy, crop, and weed) in the segmentation results. The IOU is calculated by dividing the intersection of the predicted set and ground truth by their union, as shown in (Equation 23).


(23) $$\;IOU=\frac{TP}{TP+FP+FN}.$$

The MIOU is a measure that calculates the average of the IOU values for three classes: crop, weed, and soil or paddy. It is described as (Equation 24).


(24) $$\;MIOU=\frac{IOU_{crop}+IOU_{weed}+IOU_{soil\vee paddy}}{3}.$$

The precision is a metric that measures the accuracy of a model’s positive predictions. It calculates the proportion of correctly identified positive samples (TP) out of all samples that the model classified as positive. The Precision is given in (Equation 25).


(25) $$Precision=\frac{TP}{TP+FP}.$$

The recall represents the probability of correctly identifying positive samples among the actual positive instances, as shown in (Equation 26).


(26) $$Recall=\frac{TP}{TP+FN}.$$

The F1score is calculated as the harmonic mean of precision and recall, with equal weights assigned to each of them, as shown in (Equation 27).


(27) $$\;F1score=\frac{2\times Precision\times Recall}{Precision+Recall}.$$

There is a commutative relationship between the F1score and the IOU, as shown in (Equation 28). Thus the F1score provides a direct measure of the network’s segmentation accuracy.


(28) $$IOU=\frac{F1score}{2-F1score}.$$

### 4.5 Testing on the BoniRob dataset

#### 4.5.1 Ablation studies

In this paper, we adopt a baseline using the six-stage U-structure (U_VI_) with channel numbers of {8,16,24,32,48,64} and incorporate an element-wise addition skip connection between the encoder and decoder. Single-module ablation studies are conducted on U_VI_: U_VI_+RDC, where RDC replaces the 2D convolution in stages 4-6 of the encoder; U_VI_+DA, which adds the DA before the 2D convolution in stages 2-6 of the encoder; U_VI_+SCA, incorporating the SCA into the skip connection between the encoder and decoder. Multi-module ablation studies are conducted by extending the single module as follows: U_VI_+RDC+DA, which builds upon U_VI_+RDC by adding the DA before the 2D convolution in stages 2-3 of the encoder, and before the RDC in stages 4-6 of the encoder; U_VI_+RDC+DA+ SCA further incorporating the SCA into the skip connection between the encoder and decoder, based on U_VI_+RDC+DA. Ablation studies on the Rice Seedling dataset (in Subsection 4.6.1) and the WeedMap dataset (in Subsection 4.7.1) are also conducted in the same manner.

**Table 1** shows how different modules impact the accuracy and complexity of segmentation on the BoniRob dataset, with green undertones indicating crop aspects, red undertones indicating weed aspects and bold text indicates the best results for each indicator. The settings for the following tables (**Tables 2**–**6**) are also the same. In the single-module ablation studies, the RDC enhances the receptive field, improving the ability to extract weeds from the overall target, while reducing the number of parameters and complexity. The DA reinforces the relationship between the sample and the whole, thereby improving the recall of crops and weeds and leading to more accurate segmentation targets. The SCA enhances the integration of spatial information, thereby improving the overall accuracy of image segmentation. Importantly, this enhancement is achieved without significantly increasing the number of parameters or the computational complexity of the model. In the multi-module ablation studies, the combination of RDC and DA directs the encoder’s focus toward identifying small weed targets, thereby enhancing the classification accuracy of weeds. Additionally, the inclusion of the SCA in the encoder-decoder hopping connection ensures more precise boundary delineation of crops and weeds, effectively improving overall segmentation accuracy.

**Table 1:** Evaluation results of various modules on the BoniRob dataset.

NETWORKS	Soil IOU	Crop IOU	Weed IOU	MIOU	Params (M)	FLOPs (G)	Precision	Recall	F1score
U_VI_	0.9956	0.9323	0.6955	0.8744	0.1072	0.1219	0.9688	0.9611	0.9649
							**0.9023**	0.7521	0.8204
U_VI_+RDC	0.9957	0.9385	0.7476	0.8939	**0.0649**	**0.1063**	**0.9751**	0.9616	0.9682
							0.8825	0.8302	0.8555
U_VI_+DA	0.9956	0.9364	0.7434	0.8918	0.1496	0.2467	0.9695	**0.9649**	0.9671
							0.8628	0.843	0.8528
U_VI_+SCA	0.9956	0.9366	0.7458	0.8927	0.1073	0.1305	0.9708	0.9638	0.9673
							0.8734	0.8362	0.8544
U_VI_+RDC +DA	0.9957	0.9366	0.7503	0.8942	0.1073	0.2312	0.9742	0.9604	0.9672
							0.8784	0.8374	0.8574
U_VI_+RDC +DA+SCA	**0.9957**	**0.9390**	**0.7600**	**0.8982**	0.1074	0.2397	0.9725	0.9645	0.9685
							0.8902	**0.8387**	**0.8636**

Bold text indicates the best results for each indicator.

**Table 2:** Comparison results between the proposed method and the SOTA methods on the BoniRob dataset.

NETWORKS	Soil IOU	Crop IOU	Weed IOU	MIOU	Precision	Recall	F1score	TP	FN	FP	TN
DeepLab V3+	0.9940	0.9121	0.6101	0.8388	0.9526	0.9555	0.9540	5,255,540	244,749	261,573	119,273,145
					0.8220	0.7030	0.7579	455,194	192,341	98,514	124,288,958
SEGNet	0.9952	0.9210	0.6665	0.8609	0.9444	0.9738	0.9589	5,356,167	144,122	315,558	119,219,160
					0.8587	0.7486	0.7999	484,733	162,802	79,732	124,307,740
ENet	0.9947	0.9259	0.6931	0.8713	0.9678	0.9554	0.9615	5,254,899	245,390	174,878	119,359,840
					0.8712	0.7722	0.8187	500,021	147,514	73,893	124,313,579
UNet	0.9949	0.9289	0.6740	0.8659	0.9491	**0.9776**	0.9631	**5,377,154**	**123,135**	288,333	119,246,385
					**0.9106**	0.7217	0.8052	467,335	180,200	**45,837**	**124,341,635**
UNeXt	0.9953	0.9288	0.6996	0.8746	0.9668	0.9593	0.9631	5,276,669	223,620	181,124	119,353,594
					0.8037	**0.8439**	0.8233	**546,468**	**101,067**	133,479	124,253,993
MFRWF-CWF	**0.9959**	**0.9395**	0.7554	0.8970	0.9716	0.9660	**0.9688**	5,313,665	186,624	155,314	119,379,404
					0.8784	0.8436	0.8607	546,274	101,261	75,636	124,311,836
Ours	0.9957	0.9390	**0.7600**	**0.8982**	**0.9725**	0.9645	0.9685	5,305,290	194,999	**149,851**	**119,384,867**
					0.8902	0.8387	**0.8636**	543,074	104,461	67,004	124,320,468

Bold text indicates the best results for each indicator.

**Table 3:** Evaluation results of various modules on the Rice Seedling dataset.

NETWORKS	Paddy IOU	Crop IOU	Weed IOU	MIOU	Params (M)	FLOPs (G)	Precision	Recall	F1score
U_VI_	0.9447	0.6610	0.7566	0.7874	0.1072	0.1219	0.6999	**0.9224**	0.7959
							0.8292	0.8962	0.8614
U_VI_+RDC	0.9504	0.6793	0.7576	0.7957	**0.0649**	**0.1063**	0.7509	0.8769	0.8090
							0.8367	0.8890	0.8621
U_VI_+DA	**0.9505**	0.6784	0.7585	0.7958	0.1496	0.2467	**0.7665**	0.8550	0.8083
							0.8188	**0.9114**	0.8626
U_VI_+SCA	0.9485	0.6712	0.7742	0.7980	0.1073	0.1305	0.7491	0.8658	0.8032
							0.8427	0.905	0.8728
U_VI_+RDC +DA	0.9503	0.6798	0.7632	0.7977	0.1073	0.2312	0.7525	0.8755	0.8093
							0.8297	0.9049	0.8657
U_VI_+RDC +DA+SCA	0.9492	**0.6799**	**0.7797**	**0.8030**	0.1074	0.2397	0.7401	0.8932	**0.8095**
							**0.8445**	0.9104	**0.8762**

Bold text indicates the best results for each indicator.

**Table 4:** Comparison results between the proposed method and the SOTA methods on the Rice Seedling dataset.

NETWORKS	Paddy IOU	Crop IOU	Weed IOU	MIOU	Precision	Recall	F1score	TP	FN	FP	TN
DeepLab V3+	0.9446	0.6581	0.7746	0.7925	0.7221	0.8813	0.7938	6,417,761	864,695	2,469,518	74,297,946
					**0.8679**	0.8782	0.8730	3,239,109	449,317	**493,203**	**79,868,291**
SEGNet	0.9490	0.6701	0.7501	0.7897	0.7447	0.8700	0.8024	6,335,537	946,919	2,171,939	74,595,525
					0.8062	**0.9151**	0.8572	**3,375,356**	**313,070**	811,207	79,550,287
ENet	0.9464	0.6652	0.7588	0.7901	0.7363	0.8733	0.7990	6,359,859	922,597	2,278,118	74,489,346
					0.8207	0.9095	0.8628	3,354,788	333,638	733,158	79,628,336
UNet	**0.9515**	0.6739	0.7492	0.7915	**0.7664**	0.8481	0.8052	6,176,299	1,106,157	**1,882,912**	**74,884,552**
					0.8205	0.8961	0.8566	3,305,335	383,091	723,142	79,638,352
UNeXt	0.9478	0.6712	0.7743	0.7978	0.7287	**0.8947**	0.8033	**6,515,517**	**766,939**	2,425,241	74,342,223
					0.8447	0.9029	0.8728	3,330,186	358,240	612,256	79,749,238
MFRWF-CWF	0.9504	**0.6863**	0.7678	0.8015	0.7528	0.8859	**0.8140**	6,451,810	830,646	2,118,250	74,649,214
					0.8499	0.8882	0.8686	3,276,194	412,232	578,657	79,782,837
Ours	0.9492	0.6799	**0.7797**	**0.8030**	0.7401	0.8932	0.8095	6,504,681	777,775	2,284,200	74,483,264
					0.8445	0.9104	**0.8762**	3,357,934	330,492	618,419	79,743,075

Bold text indicates the best results for each indicator.

**Table 5:** Evaluation results of various modules on the WeedMap dataset.

NETWORKS	Soil IOU	Crop IOU	Weed IOU	MIOU	Params (M)	FLOPs (G)	Precision	Recall	F1score
U_VI_	0.9722	0.7254	0.6171	0.7716	0.1072	0.1219	**0.8566**	0.8257	0.8409
							0.8157	0.7171	0.7632
U_VI_+RDC	0.9722	0.7298	0.6265	0.7762	**0.0649**	**0.1063**	0.8157	0.8739	0.8438
							0.7890	0.7527	0.7704
U_VI_+DA	0.9728	0.7278	0.6329	0.7779	0.1496	0.2467	0.8457	0.8392	0.8424
							0.7975	0.7541	0.7752
U_VI_+SCA	0.9729	0.7324	0.6354	0.7802	0.1073	0.1305	0.8424	0.8488	0.8456
							**0.8215**	0.7371	0.777
U_VI_+RDC +DA	0.9727	0.7270	0.6428	0.7809	0.1073	0.2312	0.8099	**0.8766**	0.8420
							0.7634	**0.8028**	0.7826
U_VI_+RDC +DA+SCA	**0.9730**	**0.7330**	**0.6511**	**0.7857**	0.1074	0.2397	0.8328	0.8595	**0.8460**
							0.7793	0.7984	**0.7887**

Bold text indicates the best results for each indicator.

**Table 6:** Comparison results between the proposed method and the SOTA methods on the WeedMap.

NETWORKS	Soil IOU	Crop IOU	Weed IOU	MIOU	Precision	Recall	F1score	TP	FN	FP	TN
DeepLab V3+	0.9557	0.6213	0.5432	0.7067	0.7085	0.8347	0.7664	927,270	183,679	381,583	19,243,468
					0.7015	0.7065	0.7040	479,327	199,096	203,950	19,853,627
SEGNet	0.9654	0.6519	0.4783	0.6986	0.7524	0.8300	0.7893	922,040	188,909	303,455	19,321,596
					0.7258	0.5838	0.6471	396,047	282,376	149,643	19,907,934
ENet	0.9693	0.7125	0.6162	0.7660	0.8155	0.8494	0.8321	943,642	167,307	213,545	19,411,506
					0.7586	0.7664	0.7625	519,962	158,461	165,423	19,892,154
UNet	0.9715	0.7244	0.6252	0.7737	0.8060	**0.8774**	0.8402	**974,761**	**136,188**	234,663	19,390,388
					0.7741	0.7647	0.7694	518,765	159,658	151,372	19,906,205
UNeXt	0.9707	0.7213	0.6355	0.7758	0.8413	0.8348	0.8381	927,449	183,500	174,914	19,450,137
					0.7338	**0.8258**	0.7771	**560,255**	**118,168**	203,227	19,854,350
MFRWF-CWF	**0.9732**	**0.7353**	0.6345	0.7811	**0.8514**	0.8437	**0.8475**	937,328	173,621	**163,656**	**19,461,395**
					**0.7982**	0.7558	0.7764	512,757	165,666	**129,644**	**19,927,933**
Ours	0.9730	0.7330	**0.6511**	**0.7857**	0.8328	0.8595	0.8460	954,883	156,066	191,679	19,433,372
					0.7793	0.7984	**0.7887**	541,629	136,794	153,403	19,904,174

Bold text indicates the best results for each indicator.

For crops, the proposed method exhibits a slight improvement in both Precision and Recall. This improvement can be attributed to the large size of crop targets and the low presence of weed infestation in the BoniRob dataset, demonstrating enhanced performance in segmenting crop textures and boundaries. For weeds, our method demonstrates a minor decline in Precision but a substantial increase in Recall. This discrepancy can be attributed to the small and dispersed nature of weed targets in the BoniRob dataset, wherein the greater Recall value suggests a more comprehensive weed detection. These results demonstrate that the proposed method’s effectiveness in accurately segmenting crops and weeds, even in environments with small and dispersed weed clusters.

#### 4.5.2 Comparisons between the proposed method and the SOTA methods

To assess the effectiveness of the proposed method, we conducted a series of comparative experiments using various networks, including DeepLab V3+, SEGNet, ENet, and UNet, which are commonly used in the field. In addition, we conducted comparisons with two other networks: MFRWF-CWF, specifically designed for agronomic image segmentation, and UNeXt, a lightweight network designed for real-time segmentation. Comparative experiments on the Rice Seedling dataset (in Subsection 4.6.1) and the WeedMap dataset (in Subsection 4.7.1) are also conducted in the same manner.

**Table 2** presents the comparison results between the proposed method and the SOTA methods on the BoniRob dataset, and displays the comparison results of the numbers of true positives (TP), false negatives (FN), false positives (FP), and true negatives (TN) between different methods (The total number of pixels is 125,035,007). In the case of crops, MFRWF-CWF produces more true positives, whereas the proposed method has the fewest false positives. However, the proposed method exhibits higher false negatives compared to MFRWF-CWF, resulting in a lower Recall. Additionally, although the proposed method has the highest Precision, the F1score is lower than that of MFRWF-CWF. Consequently, the crop IOU of the proposed method is slightly lower than MFRWF-CWF. In the case of weeds, the Precision value and the Recall value of the proposed method are lower than those of UNet and UNeXt, respectively. This disparity arises because UNet exhibits the fewest false positives for weeds and more true positives than the proposed method, leading to a higher precision. UNeXt achieves the most true positives and the fewest false negatives, resulting in the highest Recall. The F1score for weeds in the proposed method surpasses that of other methods, and the F1score for crops is slightly lower than that of MFRWF-CWF. Nevertheless, the MIOU of the proposed method is the highest. It indicates that our method offers better overall segmentation accuracy in scenarios where weeds are small and dispersed.

**Figure 6:**
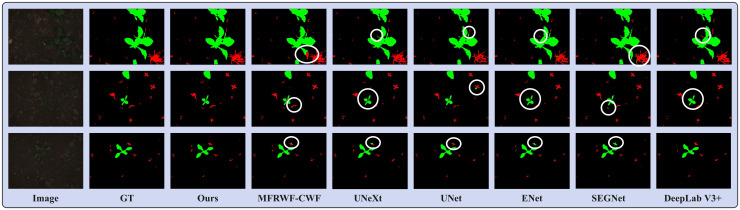
Segmentation results obtained by the proposed method and the SOTA methods on the BoniRob dataset.

**Figure 6** shows three groups of segmentation results obtained by the proposed method and the SOTA methods. Green, red, and black represent crop, weed, and soil, respectively. The white circular areas represent regions with low segmentation accuracy. as depicted in **Figure 6**), crops and weeds in the original images exhibit a high degree of separateness, with a relatively low level of weed infestation. However, weed targets are small and scattered, which poses challenges for image segmentation.

From the segmentation results, the proposed method achieves highly accurate detection of fine weeds and closely resembles the Ground Truth (GT). In contrast, the SOTA methods often misclassifies fine weed targets as crops, especially ENet and SEGNet. Additionally, UNet tends to omit weeds. UNeXt and DeepLab V3+ exhibited rougher plant boundary processing, leading to misclassification of crop internals as soil background.

### 4.6 Testing on the Rice Seedling dataset

#### 4.6.1 Ablation studies

Evaluation results of various modules on the Rice Seedling dataset are presented in **Table 3**. In the single-module ablation studies, the RDC improves the segmentation accuracy of crops under occlusion, while reducing the number of parameters and complexity. The DA strengthens the ability to recognize plant reflections in the background, effectively improving the anti-interference ability of plant reflections, and subsequently enhancing the segmentation accuracy of the background. The SCA significantly improves the segmentation accuracy of weeds with only a minimal increase in parameter number and complexity. In the multi-module ablation studies, the combination of RDC and DA enhances the information fusion capacity across multiple channels, ensuring a balanced trade-off between Precision and Recall for segmentation targets, ultimately leading to improved overall segmentation performance. Moreover, incorporating the SCA into the encoder-decoder hopping connection enables the network to prioritize crop and weed boundaries, resulting in a significant improvement in pixel classification accuracy for crops and weeds in overlapping regions.

For crops, the proposed method has improved Precision while decreasing Recall. This signifies an enhanced ability to accurately classify the target crop in scenarios with overlapping crops and weeds, effectively mitigating the impact of plant reflections in the background. For weeds, both the Precision and Recall are improved by the proposed method. This suggests that the target weeds can be comprehensively and accurately segmented even in weed-infested environments.

#### 4.6.2 Comparisons between the proposed method and the SOTA methods

**Table 4** presents the comparison results between the proposed method and the SOTA methods on the Rice Seedling dataset, and gives the comparison results of the numbers of TP, FN, FP, and TN between different methods (The total number of pixels is 84,049,920). In the case of crops, the proposed method yields more true positives and fewer false negatives, resulting in a higher Recall than MFRWF-CWF. MFRWF-CWF exhibits fewer false positives. Consequently, the average crop Precision and Recall of MFRWF-CWF are slightly higher than those of our method. In the case of weeds, the weeds Precision and Recall of the proposed method are lower than DeepLab V3+ and SEGNet, respectively. DeepLab V3+ produces the least number of false positives, resulting in higher Precision than the proposed method. On the other hand, SEGNet generates the highest number of true positives and the least number of false negatives, leading to the highest Recall among all methods. The F1score of weeds obtained by the proposed method is the highest. Moreover, the MIOU given by the proposed method is also the highest. It demonstrates that our method outperforms other comparison methods in terms of overall segmentation accuracy even in environments with complex background disturbances, overlapping, and occlusion of crops and weeds.

**Figure 7:**
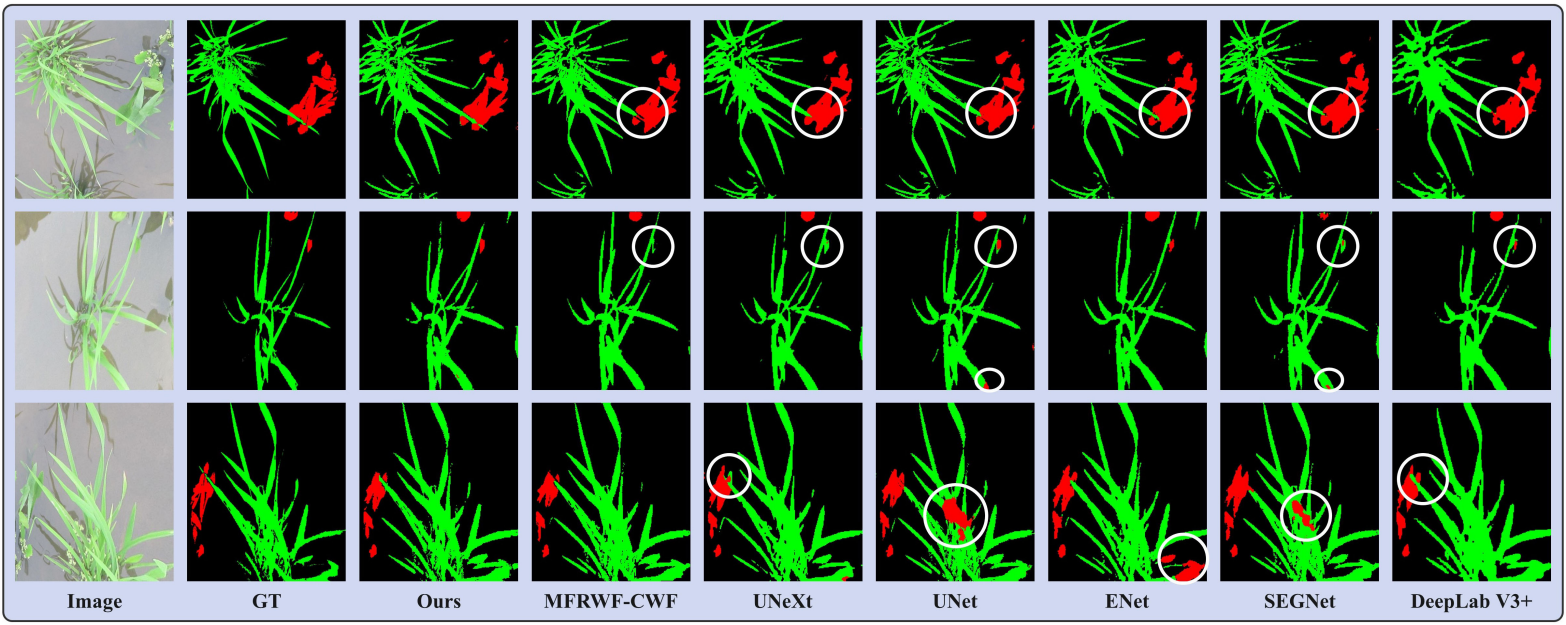
Segmentation results obtained by the proposed method and the SOTA methods on the Rice Seedling dataset.

**Figure 7** shows three groups of segmentation results obtained by the proposed method and the SOTA methods. Green, red, and black represent crop, weed, and soil, respectively. The white circular areas represent regions with low segmentation accuracy. In the Rice Seedling dataset, as depicted in **Figure 7**, the background is paddy. The reflection of plants in the water causes interference in the segmentation task. Additionally, there is a minor overlap and occlusion of crops, as well as a significant infestation of weeds. Therefore, it is crucial to enhance the anti-interference ability towards reflection and accurately delineate the boundaries of crops and weeds to improve segmentation results.

From the segmentation results, it can be seen that the proposed method outperforms others in correctly identifying and classifying weeds, especially in situations where a few weeds are obscured by the crop. Additionally, our method exhibits the highest similarity to the GT, whereas the SOTA methods faces challenges in accurately classifying crops when they are occluded by weeds. MFRWF-CWF and UNeXt have misclassified small weed targets, whereas UNet, ENet, and SEGNet have incorrectly classified extensive crop areas. Moreover, DeepLab V3+ exhibits limitations in capturing fine plant texture details.

### 4.7 Testing on the WeedMap dataset

#### 4.7.1 Ablation studies

Evaluation results of various modules on the WeedMap dataset are presented in **Table 5**. In the singlemodule ablation studies, the RDC enhances the detection of tiny targets, improving the segmentation performance for both crops and weeds, while reducing the number of parameters and complexity. The DA strengthens the network’s ability to learn the overarching patterns within the dataset, leading to a significant enhancement in weed segmentation accuracy and the integration of multi-channel information. Moreover, it contributes to the improved accuracy of target segmentation. The SCA strengthens the fusion of spatial information, leading to a substantial enhancement in the segmentation accuracy of crops, with only a negligible increase in the number of parameters and complexity. In the multi-module ablation studies, the combination of RDC and DA focuses the encoder on recognizing tiny targets, significantly improving the Recall of segmented targets. Furthermore, incorporating the SCA into the encoder-decoder connection not only enhances the accuracy of segmenting crop and weed boundaries but also improves the overall segmentation performance of the network.

For crops and weeds, the proposed method has improved Recall while decreasing Precision. The decrease in Precision can be attributed to the regularity of crop generating regions in the WeedMap dataset, but the more chaotic distribution of weeds with high weed infestation. The improvement in Recall indicates an improvement in the ability to comprehensively detect segmentation targets with small crop and weed targets. Ultimately, the overall segmentation accuracy is improved by increasing the comprehensiveness of detection. These results show that in segmenting aerial images captured by UAVs with small targets, the method maintains stable and accurate results despite the presence of high levels of weed infestation.

#### 4.7.2 Comparisons between the proposed method and the SOTA methods

**Table 6** presents the comparison results between the proposed method and the SOTA methods on the WeedMap dataset, and gives the comparison results of the numbers of TP, FN, FP, and TN between different methods (The total number of pixels is 20,736,000). In the case of crops, the proposed method gives a higher number of true positives and a lower number of false negatives comparing to MFRWF-CWF. Consequently, the Recall value of our method is superior to that of MFRWF-CWF. However, MFRWF-CWF yields the minimum number of false positives, leading to a higher precision. Following the reconciliation of precision and recall through the F1score, it is found that the F1score achieved by the proposed method is slightly lower than that of MFRWF-CWF, ultimately resulting in a slightly lower crop IOU compared to MFRWF-CWF. In the case of weeds, the proposed method yields a higher number of true positives. Conversely, MFRWF-CWF produces the fewest false positives, leading to a higher precision compared to the proposed method. UNeXt achieves the highest recall by yielding the most true positives and the fewest false negatives. The F1score of obtained by the proposed method weeds is higher than that of other methods. The F1score of crops obtained by the proposed method is slightly lower than that of MFRWF-CWF. Nevertheless, the proposed method achieves the highest MIOU. This highlights that the proposed method exhibits significantly superior overall segmentation accuracy in UAV overhead images characterized by smaller targets and high levels of overlap.

**Figure 8:**
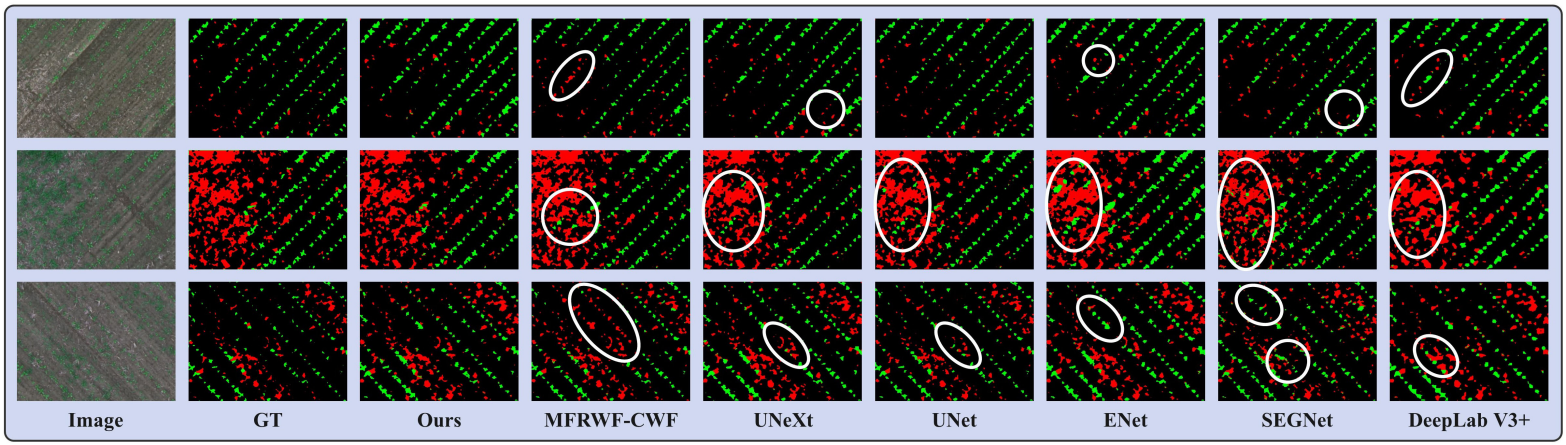
Segmentation results obtained by the proposed method and the SOTA methods on the WeedMap dataset.

**Figure 8** shows three groups of segmentation results obtained by the proposed method and the SOTA methods. Green, red, and black represent crop, weed, and soil, respectively. The white circular areas represent regions with low segmentation accuracy. The WeedMap dataset comprises aerial images captured by a UAV, as depicted in **Figure 8**, with the primary segmentation task focusing on identifying small targets. Although the growth area of the crops appears uniform, the weeds grow in a disorderly manner, which makes it challenging to accurately classify them. Additionally, the crops and weeds often overlap, further complicating the segmentation process. Therefore, the key to improving the segmentation effectiveness lies in the ability to detect and classify these small targets correctly.

From the segmentation results, the proposed method demonstrates superior segmentation accuracy in the region where crops and weeds overlap. Additionally, our method exhibits the highest similarity to the GT, whereas the SOTA methods is prone to misclassify weeds that grow in the crop area, especially UNet and ENet, MFRWF-CWF has a tendency to misclassify the crop as a weed. UNeXt and SEGNet display weaknesses in detecting tiny weeds, which can lead to pixel loss. Additionally, ENet and DeepLab V3+ exhibit coarse plant boundary processing, making it challenging to distinguish the soil background when there is a high weed infestation.

### 4.8 Complexity comparison of different algorithms

**Table 7** presents the complexity comparison between the proposed method and SOTA methods in terms of the number of parameters, FLOPs, inference time and FPS. The best results for each metric are highlighted in bold. In **Table 7**, it can be observed that the proposed method boasts the smallest number of model parameters, only 0.11M. This figure represents a remarkable reduction of 96.5% compared to the SOTA method MFRWF-CWF, a reduction of 92.5% when compared to the lightweight U-network UneXt, and a reduction of 68.6% compared to the generic lightweight network Enet. The significant reduction in parameter count demonstrates the efficiency of the proposed method. In terms of the number of FLOPs, the proposed method excels once more, with a mere 0.24G. This figure is the lowest among all the compared methods, indicating a lower algorithmic complexity.

**Table 7:** Comparisons of the number of parameters, Floating-point Operations(FLOPs), inference time and Frame Per Second(FPS).

NETWORKS	Params (M)	FLOPs (G)	Inference time (msec)	FPS
DeepLab V3+	40.37	11.71	46.52	21.50
SEGNet	29.44	40.20	152.23	6.57
ENet	0.35	0.55	69.23	14.44
UNet	13.40	31.13	123.69	8.08
UNeXt	1.47	0.58	21.39	46.74
MFRWF-CWF	3.14	24.00	74.97	13.33
Ours	**0.11**	**0.24**	**18.14**	**55.10**

The best results for each metric are highlighted in bold.

Since the proposed network is primarily designed for intelligent weeding robots, computational operations are executed in the form of on-board computation (edge computation) on the embedded system inside the mobile robot when calculating the inference time and FPS. We use an NVIDIA Jetson Xavier NX for measuring these metrics, which utilizes GPUs with 384 CUDA cores. Inference framework in NVIDIA Volta. The central processing unit (CPU) shares 8Gb of memory with the GPU, and the memory bandwidth is 59.7GB/s. The power usage is 10W. As presented in **Table 7**, the proposed method takes 18.14 msec to process an image, and the FPS is 55.10 frames/sec, surpassing all compared methods. Therefore, it is evident that the proposed method is suitable for application in embedded systems with limited hardware resources.

## 5 Discussion

The current methods based on CNNs for crop and weed semantic segmentation can be categorized into two types: single-CNN structure and multi-CNN structure. The multi-CNN structure divides the task into stages, which improves segmentation performance to some extent. However, due to the independent nature of the model task, these methods incur a high computational cost and increase inference time in multiples. As a result, they are not suitable for implementation on mobile intelligent robots for crop and weed segmentation. The single-CNN structure is more suitable for embedded systems compared to the multi-CNN structure. Therefore, the single-CNN structure is selected in our proposed method. In different practical applications, the structure of CNNs can vary. The MFRWF-CWF network is specifically designed for agronomic image segmentation, it is different from other general-purpose networks such as DeepLab V3+, SEGNet, ENet, and UNet. Additionally, the UNeXt network is a lightweight network that is specifically designed for real-time segmentation.

The MFRWF-CWF network utilizes a lightweight backbone with restricted kernel convolution dimensions. The basic block contains Resnet34 iterative parameters, and a feature reweighting module based on multilevel attention is employed to eliminate background clutter and fine-tune the target signal. Furthermore, fusion takes place between each weighted feature map and all other features to enhance the background signal of crop and weed. Ultimately, the MFRWF-CWF decoder combines all object maps to maintain rich contextual information. Results displayed in **Tables 2**, **4**, **6** for the Bonirob dataset, Rice Seedling dataset and WeedMap dataset reveal that the MFRWF-CWF technique achieves superior performance in terms of MIOU and F1score compared to other general-purpose networks. However, it is important to note that MFRWF-CWF incurs an overhead of 3.14M (as shown in **Table 7**) during training, which is approximately 9 times larger than the training parameters of ENet, a general-purpose lightweight network. Additionally, MFRWF-CWF has significantly higher FLOPs than Enet. Its inference time and FPS are less favorable when compared to other general-purpose networks. UneXt adopts a convolutional and MLP-based architecture, incorporating a tokenized MLP block with shifted MLPs for labeling and projecting convolutional features. The results shown in **Table 7** demonstrate that UneXt slightly outperforms the general-purpose lightweight network ENet in terms of training parameters and FLOPs, and significantly outperforms the other networks in terms of inference time and FPS. However, when considering the segmentation results showcased in **Tables 2**, **4**, **6**, UneXt does not possess an advantage in crop and weed segmentation tasks comparing to MFRWF-CWF. Therefore, the proposed method in the paper aims to address the challenge of designing a network that achieves improved inference time and FPS while maintaining competitive segmentation performance.

The method proposed in this paper utilizes a six-stage U-shaped structure as the baseline. To further reduce network parameters, we choose to reduce the output feature map and adjust the number of channels to {8,16,24,32,48,64}. Moreover, we employ element-wise addition instead of concatenation to decrease the number of convolution operations in the encoder-decoder skip connection. Although this reduction in parameters and complexity initially hampers the baseline’s feature extraction capability, leading to inadequate segmentation results that fail to meet actual requirements, we address this issue by introducing a lightweight module to enhance segmentation accuracy. Since the crop and weed dataset has a relatively small number of samples and some images possess only a single feature, the crop and weed categories are imbalanced, rendering the training process susceptible to overfitting. To overcome this problem, we propose the DA, which improves the network model’s ability to learn information from the entire dataset, thereby serving as a regularization technique to enhance generalization. DA significantly alleviates the overfitting issue during training. Additionally, it facilitates the integration of cross-channel inter-channel information, strengthens the utilization of channel attention, and ultimately improves segmentation performance. To tackle the challenges associated with classifying semantic information of crops and weeds, we propose the RDC to replace 2D convolution in deep-level networks. RDC integrates global information and local information, enhances the computational efficiency of dilated convolution, and reduces the number of network parameters. Furthermore, we introduce the SCA to mitigate the loss of spatial information caused by the superposition of convolutional layers. The output feature maps of the encoders at each stage are processed using shared weights, which further simplifies the model and obtains feature maps containing spatial attention weights that are combined with the decoder. The segmentation results, as shown in **Tables 2**, **4**, **6**, demonstrate that our method achieves competitive MIOU scores compared to other approaches. Although our method may exhibit lower IOU on a single target (soil or paddy, crops) compared to other methods, it still proves effective overall.

We conducted comparative experiments with the SOTA methods on three distinct datasets: the BoniRob dataset, which covers soil crops; the Rice Seedling dataset, which involves paddy crops; and the WeedMap dataset, which consists of UAV aerial views. These datasets provide diverse scenarios and test the generalization ability of the network. The metrics data presented in **Tables 2**, **4**, **6**, along with the segmentation results depicted in **Figures 6**–**8**, reveal that the BoniRob dataset exhibits decentralized crop and weed distributions, with the lowest level of weed infestation among the three datasets. Notably, this paper’s method slightly falls behind the MFRWF-CWF in terms of background IOU and crop IOU, indicating the potential for improvement in processing crop texture details. The accuracy of overall segmentation is mainly affected by the smaller size of the weed target. However, the method proposed in this paper outperforms other SOTA methods in terms of weed IOU, resulting in a better MIOU. In the Rice Seedling dataset, overlapping and shading between crops and weeds occur, especially in areas with high weed infestation. Moreover, the presence of aquatic crops introduces reflection on the water surface, making the background complexity the highest among the three datasets. In terms of background IOU, the method presented in this paper performs lower than UNet and MFRWF-CWF, suggesting room for improvement in handling complex background interference. Although this paper’s method also exhibits a slightly lower crop IOU compared to MFRWF-CWF, it surpasses other SOTA methods in terms of weed IOU. resulting in a better MIOU. The WeedMap dataset includes aerial images captured by UAVs, resulting in a small overall segmentation target and a chaotic weed growth area where crops and weeds frequently overlap. Consequently, this dataset exhibits the highest level of weed infestation compared to the other two datasets. Similar to the previous cases, this paper’s method shows slightly lower performance in terms of background IOU and crop IOU when compared to MFRWF-CWF. This highlights the need for improvement in delineating plant boundaries. The inability to accurately detect and categorize tiny weed targets within the overlapping region of crops and weeds remains the main factor affecting overall segmentation accuracy, the method proposed in this paper excels in weed IOU, surpassing other SOTA methods. This demonstrates its superiority in environments with severe weed infestation, leading to a better MIOU. Although our method may exhibit lower IOU on a single target (soil or paddy, crops) compared to other methods, it still proves effective overall. Moreover, the proposed method excels in terms of lightweight, as demonstrated in **Table 7**. The proposed method outperforms the SOTA methods in terms of the number of model parameters and the number of FLOPs, indicating its lower training cost and network complexity. Furthermore, when considering inference time and FPS based on NVIDIA Jetson Xavier NX, the proposed method surpasses the general-purpose lightweight network ENet and the real-time segmentation lightweight network UNeXt. This suggests that the proposed method is highly suitable for deployment on embedded systems with constrained computational resources, making it an ideal choice for semantic segmentation tasks involving crops and weeds in the context of intelligent weeding robots operating in resource-constrained environments.

In summary, the segmentation performance of the method proposed in this paper is competitive and outperforms other SOTA methods in terms of model parameters, FLOPs, inference time and FPS. This indicates that the proposed method can be implemented on intelligent weeding robots with limited hardware resources, ensuring both lower hardware resource utilization and competitive segmentation performance. However, it is worth noting that the segmentation accuracy for some targets is relatively low, as depicted in **Tables 2**, **4**, **6**. These tables show cases where the IOU scores for crops are lower than those obtained by MFRWF-CWF on the BoniRob dataset, Rice Seedling dataset and WeedMap dataset. Consequently, there is still room for further improvement in segmentation performance while maintaining a low number of parameters and complexity in the network.

## 6 Conclusions

Existing semantic segmentation networks for crops and weeds primarily focus on enhancing segmentation performance while disregarding their practical applications in intelligent weeding robots. Hence, this paper proposes an attention-aided lightweight network for crop and weed semantic segmentation, aiming to improve inference time and FPS without compromising segmentation performance. Firstly, we implement regularization and integrate channel information to address the issue of overfitting during training due to the insufficient number of samples in the crop and weed dataset. It enhances the attention mechanism’s ability to generalize and enables the network model to learn information more efficiently from the entire dataset. Subsequently, the integration of local and global information is employed to enhance the classification of semantic information related to crops and weeds, while also significantly reducing the number of network parameters. Next, enhancements are made to acquire spatial information effectively to minimize the loss of spatial details caused by convolution layers’ overlapping. Furthermore, the network’s complexity is reduced by adopting a shared weights approach instead of a complex connection between the encoder and decoder. Finally, these modules are combined with a lightweight baseline. Comparative experimental and visualization results on publicly available datasets, combined with test results on embedded systems, demonstrate that the proposed method successfully achieves a robust balance between network complexity and segmentation performance.

Lightweighting is essential for a method to be deployed in smart weeding robot and perform well, given the limited hardware resources of these robots. This study aims to provide valuable insights into lightweight semantic segmentation of crops and weeds. Additionally, the experiments conducted in this paper demonstrate that the proposed method achieves competitive segmentation performance while efficiently operating within the hardware limitations of intelligent weeding robots.

Nevertheless, the segmentation accuracy for certain targets remains inadequate. In the future, we will focus on further reducing the parameter count and complexity of the network, improving inference time and FPS for embedded systems, and enhancing segmentation accuracy.

## Data availability statement

The original contributions presented in the study are included in the article/supplementary material. Further inquiries can be directed to the corresponding author.

## Author contributions

YW: Conceptualization, Formal analysis, Investigation, Methodology, Writing – original draft, Writing – review & editing. YF: Funding acquisition, Investigation, Methodology, Writing – review & editing, Formal analysis. XZ: Funding acquisition, Software, Validation, Visualization, Writing – review & editing. GW: Funding acquisition, Software, Validation, Visualization, Writing – review & editing.
